# The Claudin-like Protein HPO-30 Is Required to Maintain LAChRs at the *C. elegans* Neuromuscular Junction

**DOI:** 10.1523/JNEUROSCI.3487-17.2018

**Published:** 2018-08-08

**Authors:** Pallavi Sharma, Lei Li, Haowen Liu, Vina Tikiyani, Zhitao Hu, Kavita Babu

**Affiliations:** ^1^Department of Biological Sciences, Indian Institute of Science Education and Research Mohali, Knowledge City, Punjab, India and; ^2^Queensland Brain Institute, Clem Jones Centre for Ageing Dementia Research, University of Queensland, St. Lucia, Queensland 4072, Australia

**Keywords:** AChRs, HPO-30, levamisole, neuromuscular junction

## Abstract

Communications across chemical synapses are primarily mediated by neurotransmitters and their postsynaptic receptors. There are diverse molecular systems to localize and regulate the receptors at the synapse. Here, we identify HPO-30, a member of the claudin superfamily of membrane proteins, as a positive regulator for synaptic localization of levamisole-dependent AChRs (LAChRs) at the *Caenorhabditis elegans* neuromuscular junction (NMJ). The HPO-30 protein localizes at the NMJ and shows genetic and physical association with the LAChR subunits LEV-8, UNC-29, and UNC-38. Using genetic and electrophysiological assays in the hermaphrodite *C. elegans*, we demonstrate that HPO-30 functions through Neuroligin at the NMJ to maintain postsynaptic LAChR levels at the synapse. Together, this work suggests a novel function for a tight junction protein in maintaining normal receptor levels at the NMJ.

**SIGNIFICANCE STATEMENT** Claudins are a large superfamily of membrane proteins. Their role in maintaining the functional integrity of tight junctions has been widely explored. Our experiments suggest a critical role for the claudin-like protein, HPO-30, in maintaining synaptic levamisole-dependent AChR (LAChR) levels. LAChRs contribute to <20% of the acetylcholine-mediated conductance in adult *Caenorhabditis elegans*; however, they play a significant functional role in worm locomotion. This study provides a new perspective in the study of LAChR physiology.

## Introduction

Claudins are tetraspanin membrane proteins that play crucial roles in the formation and integrity of tight junctions and in regulating paracellular transport. Claudin proteins also function to regulate channel activity, intercellular signaling, and cell morphology (Turksen and Troy, 2004; Angelow et al., 2008). Expression of claudins in different organs and the deregulation of these proteins are associated with diseases, such as cancers, renal disorders, and deafness (Singh et al., 2010; Goncalves et al., 2013). In the nervous system, claudins serve as an integral component of the blood–brain barrier by regulating paracellular ion selectivity (Matter and Balda, 2003; Papadopoulos et al., 2004).

Most claudins have a PDZ binding motif at their C terminus that allows binding to the PDZ domains of cytoplasmic scaffold proteins, such as ZO-1/2/3, PATJ, and MUPP1. This association allows for claudins to form connections with the actin cytoskeleton (Itoh et al., 1999; Hamazaki et al., 2002; Sawada et al., 2003). Further, claudins are known to interact with nontight junction proteins, such as cell adhesion molecules EpCam, tetraspanin, and signaling proteins, such as ephrin A, ephrin B, and their receptors EphA and EphB (Ladwein et al., 2005; Tanaka et al., 2005a,b; Kovalenko et al., 2007; Rubinstein, 2011). The interaction of claudins with the actin cytoskeleton and with nontight junction proteins suggests that claudins might have functions other than in barrier formation. This work explores a nontight junction role for a *Caenorhabditis elegans* claudin-like protein, HPO-30.

The *C. elegans* genome encodes 18 claudins and claudin-like proteins ((Simske and Hardin, 2011) and wormbase (www.wormbase.org)). The functions of some of these claudin-like proteins have been studied. For example, CLC-1 localizes to epithelial cell junctions in the pharynx, where it regulates barrier functions (Asano et al., 2003). VAB-9, a divergent claudin-like protein, similar to vertebrate BCMP1 (brain cell membrane protein1), localizes to epithelial cell contacts and interacts with the cadherin-catenin complex during epidermal morphogenesis (Simske et al., 2003). Another protein, NSY-4, is related to the gamma subunit of voltage-gated calcium channels, TARP/Stargazin, as well as to other Claudin superfamily proteins. Previous studies have shown that the gamma subunit regulates the neuronal calcium channels and TARPs regulates AMPA-type glutamate receptor localization and activity (Heiskala et al., 2001; Arikkath and Campbell, 2003; Kang and Campbell, 2003; Van Itallie and Anderson, 2006). The *C. elegans* NSY-4 performs a dual function of regulating channel activity and adhesion function (Vanhoven et al., 2006). These functions of claudins in the regulation or recruitment of signaling proteins, cell adhesion molecules, and Ig proteins suggest important roles for claudins in the development and/or functioning of the nervous system.

To identify the function of the claudin family proteins at the *C. elegans* neuromuscular junction (NMJ), we performed an aldicarb-based screen on all available claudin mutants. We found mutants of a claudin homolog, *hpo-30*, to be extremely resistant to aldicarb. HPO-30 has previously been shown to be involved in the stabilization of dendritic branching of PVD neurons (Smith et al., 2013; O'Brien et al., 2017). Very recent studies show that HPO-30 functions through the WAVE regulatory complex and the membrane protein DMA-1 to allow for F-actin assembly and normal dendritic branching of the PVD neuron (Zou et al., 2018). Here we show that HPO-30 is required for the maintenance of levamisole sensitive AChRs (LAChRs) at the NMJ. Using genetic and electrophysiological analyses, we show that HPO-30 functions through Neuroligin/NLG-1 to maintain LAChRs at the NMJ. Together, our data implicate HPO-30 specifically in LAChR maintenance at the *C. elegans* NMJ.

## Materials and Methods

### 

#### Strains

All strains were maintained at 20°C as described previously (Brenner, 1974). The *E. coli* strain OP50 was used for seeding the *C. elegans* plates. The Bristol N2 strain was used as the wild-type (WT) control strain. The mutant strains used in this study were as follows: *hpo-30* (*ok2047*), *nlg-1* (*ok259*), *nrx-1* (*ok1649*), and *lev-8* (*ok1519*) (Jospin et al., 2009; Calahorro and Ruiz-Rubio, 2012, 2013; Hu et al., 2012; Smith et al., 2013). Further description of these alleles is available at wormbase (www.wormbase.org). All experiments in this study were performed using hermaphrodite *C. elegans*.

#### Constructs and transgenes

All constructs were generated using standard cloning procedures (Sambrook and Russell, 2001). *pPD49.26* or *pPD95.75* served as the vector backbone for all the constructs. The transcriptional reporter for *hpo-30* was made by cloning a 3 kb region upstream of the *hpo-30* start codon into the *pPD95.75* vector. A 3 kb *myo-3* promoter was used for expression of HPO-30 in the body-wall muscles, and a 1.4 kb *rab-3* promoter was used for expression of HPO-30 pan-neuronally. For all the rescue experiments, a 1.5 kb genomic fragment of *hpo-30* was cloned downstream of the various promoters. For the HPO-30 colocalization experiment, the mCherry was cloned downstream of HPO-30 in the P*myo-3*::HPO-30 plasmids. All the constructs were sequenced, and transgenic lines were generated by microinjection as described previously (Mello et al., 1991; Mello and Fire, 1995). A complete list of the primers used in this study is available in Table 1, and a complete list of the plasmids and strains used in this study is detailed in Tables 2, 3, and 4.

**Table 1.** List of primers

Primer code	Sequence	Comment	Gene
PS104	TTGGTGTGGCTCAGATTGTTC	Genotyping forward external	*hpo-30*
PS105	AGAGGACTAAACAACGAGAACGCAG	Genotyping forward internal	*hpo-30*
PS106	AATCTTGAGTGGCTCTGTTGG	Genotyping reverse external	*hpo-30*
ST223	CGCGGGATAGTGACGAAA	Genotyping forward external	*nrx-1*
ST224	CCATGCTCAACGAGAGAAGC	Genotyping forward internal	*nrx-1*
ST225	CCTCCGGCCATCAACTATC	Genotyping reverse external	*nrx-1*
ST226	GTGGATCCGTTCCGAAGA	Genotyping forward external	*nlg-1*
ST227	GAGAGCCCCTTATTCCACTG	Genotyping forward internal	*nlg-1*
ST228	GATGGACAGGTGGGTTGAAG	Genotyping reverse external	*nlg-1*
PS344	CATATGTATGTGTCTCTTGTTTCCG	Genotyping WT forward	*lev-8*
PS345	CATATGTATGTGTCTCTTGTTTCCA	Genotyping mutant forward	*lev-8*
PS346	TTGATCTGACGGAATGTGTA	Genotyping reverse	*lev-8*
PS284	AACTGCAGCAGCGTTGTGTTTCTGAAGATG	Cloning forward PstI	P*hpo-30*
PS285	CGGGATCCGTACATAATTAATGGCATTCCG	Cloning reverse BamH1	P*hpo-30*
PS402	AATTAAGCTTAGTGATTATAGTCTCTGTTTTCGTTA	Cloning forward HindIII	P*myo-3*
PS403	ACTTGTCGACCATTTCTAGATGGATCTAGTG	Cloning reverse SalI	P*myo-3*
PS453	ACTTGTCGACTTATGTACAAATTTCTATTAGTCAC	Cloning forward SalI	HPO-30
PS454	AATTACCGGTCATACTGCTGTCATCGTCAAT	Cloning reverse SalI	HPO-30
PS114	ACTTAATGGCACGGATAGACAGAAGC	Cloning forward AflII	P*rab-3*
PS115	CCGCTCGAGTAACACTTCCTAGTAGTAATGCCTC	Cloning reverse Xho I	P*rab-3*
PS130	CCGCTCGAGATGCCATTAATTATGTACA	Cloning forward Xho I	*hpo-30*
			gDNA
PS136	GGACTAGTTCACATACTGCTGTCATCGTC	Cloning reverse Spe I	*hpo-30*
			gDNA
PS434	AACTAGCTAGCATCTTTCGGTCGTTGGTACG	Cloning forward NheI	*nlg-1* RNAi
PS435	AAAACTGCAGCATTCACTTGGTTTGGGCTT	Cloning reverse PstI	*nlg-1* RNAi
PS436	AAAAACTGCAGAACAAACGTCGTGTGCATGT	Cloning forward NheI	*hpo-30* RNAi
PS437	AACTAGCTAGCTCCGTTCATTCCTCCAATTC	Cloning reverse PstI	*hpo-30* RNAi
PS304	CGGAATTCCTACTTATACAATTCATCCATGCCACC	Cloning reverse EcoRI	mCherry
PS355	AATTACCGGTATGGTCTCAAAGGGTGAAGAAG	Cloning forward Age I	mCherry
PS371	TGTTGCTCATCAATGTGGACG	Forward	*unc-29* qPCR
PS372	ACCTCGTAATTTCCATCGGCA	Reverse	*unc-29* qPCR
PS373	ACCTCGTAATTTCCATCGGCA	Forward	*unc-38*
			qPCR
PS374	ATCGCCGAACTGCTGAAAAA	Reverse	*unc-38*
			qPCR
PS398	TTGCAAAGTATCTTTTGCTCACT	Forward	*unc-63*
			qPCR
PS399	TCAGTAATGGGAGAATATCAAGGAA	Reverse	*unc-63*
			qPCR

**Table 2.** List of plasmids

Serial no.	Plasmid no.	Plasmid
1	pBAB201	P*hpo-30::*GFP
2	pBAB203	P*rab-3::hpo-30* gDNA
3	pBAB204	P*myo-3::hpo-30* gDNA
4	pBAB205	P*unc-17::hpo-30* gDNA
5	pBAB220	P*myo-3:: hpo-30* gDNA::mCherry
6	pBAB241	*nlg-1*(RNAi) in L4440
7	pBAB242	*hpo-30*(RNAi) in L4440

**Table 3.** List of integrated lines

Serial no.	Integrated line no.	Plasmid	Source and reference
1	*nuIs321*	P*unc-17::*mCherry	Josh Kaplan laboratory (Babu et al., 2011)
2	P*unc-17::*ChIEF	P*unc-17::*ChIEF	From Erik Jorgensen laboratory
5	*nuIs160*	P*unc-129::*SYD-2::GFP	Josh Kaplan laboratory (Sieburth et al., 2005)
4	*nuIs299*	P*myo-3::*ACR-16::GFP	Josh Kaplan laboratory (Babu et al., 2011)
5	*hpIs3*	P*unc-25::*SYD-2::GFP	CGC
6	*nuIs283*	P*myo-3::*UNC-49::GFP	Josh Kaplan laboratory (Babu et al., 2011)
7	*cwIs6*	CAM-1::GFP	Wayne Forrester laboratory (Kim and Forrester, 2003)
8	*IhIs6*	P*unc-25::*mCherry	Erik Lundquist (Norris and Lundquist, 2011)
9	*akIs38*	UNC-29::GFP	Villu Maricq laboratory (Francis et al., 2005)
10	*kr98::*YFP	UNC-63::YFP	Jean-Louis Bessereau laboratory (Rapti et al., 2011)
11	*kr208::*tagRFP	UNC-29::tagRFP	Jean-Louis Bassereau laboratory (Richard et al., 2013)
12	*vjIs105*	P*nlg-1::*NGL-1::GFP	Derek Sieburth laboratory (Staab et al., 2014)
13	*juIs76*	P*unc-25::*GFP	Yishi Jin laboratory (Baran et al., 2010)
14	*wyEx5333*	P*hlh-1::*NLG-1	Kang Shen laboratory (Maro et al., 2015)

**Table 4.** List of strains

Strain	Genotype	Comments	Figure(s)
BAB 206	*hpo-30*	CGC, RB1657 (outcrossed 4×)	1–8
BAB 253	*IndEx205* (*hpo-30*; P*unc17::*HPO-30)		1
BAB 254	*IndEx201* (P*hpo-30::*GFP)		1
BAB 208	*IhIs6; IndEx201* (P*hpo-30::*GFP)		1
BAB 211	*nuIs321; IndEx201* (P*hpo-30::*GFP)		1
BAB 252	*IndEx204* (*hpo-30*; P*myo-3::*HPO-30)		1, 4, and 5
BAB 251	*IndEx203* (*hpo-30*; P*rab-3::*HPO-30)		1, 4, and 5
BAB 239	*hpo-30*; P*unc-17::*ChIEF		3
BAB 207	*hpo-30*; *nuIs321*		2
BAB 205	*hpo-30*; *juIs76*		2
BAB 209	*hpo-30*; *nuIs160*		2
BAB 210	*hpo-30*; *hpIs3*		2
BAB 249	*hpo-30; nuIs299*		5
BAB 246	*hpo-30;* UNC-63::YFP		5
BAB 228	*hpo-30; akIs38*		5
BAB 242	*CwIs6*; UNC-29::tagRFP		5
BAB 243	*hpo-30; CwIs6*; UNC-29::tagRFP		5
BAB 250	*hpo-30; nuIs283*		5
BAB 232	*lev-8*	CGC, VC1041 (outcrossed 3×)	5
BAB 239	*hpo-30; lev-8*		5
BAB 256	*IndEx220 akIs38;* (P*myo-3::*HPO-30::mCherry)		6
BAB 262	*nlg-1*	CGC, VC1416 (outcrossed 3×)	7 and 8
BAB 261	*nrx-1*	CGC, VC228 (outcrossed 4×)	7 and 8
BAB 241	*nrx-1 hpo-30*		7 and 8
BAB 245	*hpo-30; nlg-1*		7 and 8
BAB 247	*nlg-1*; P*unc-17::*ChIEF		8
BAB 248	*nrx-1*; P*unc-17::*ChIEF		8
BAB 257	*hpo-30*; *viIs105*		8
BAB 275	*hpo-30;* UNC-29::tagRFP		8
BAB 276	*hpo-30; nlg-1;* UNC-29::tagRFP		8
BAB 277	*nlg-1;* UNC-29::tagRFP		8

#### Behavioral assays

##### Aldicarb assay.

Aldicarb assays were performed as described previously (Harada et al., 1994; Miller et al., 1996; Nurrish et al., 1999). Plates containing 1 mm aldicarb (Sigma-Aldrich; 33386) were prepared 1 d before the assay and allowed to dry at room temperature. Young adult animals (20–25) were picked and placed on aldicarb plates. The animals were scored for paralysis by gently prodding them after every 10 min with a pick, for up to 2 h. The assays were performed in triplicates with the experimenter being blind to the genotype of the animals. For simplicity, the percentage of animals that were paralyzed at the 100 min time point was plotted.

##### Levamsiole assay.

Tetramisole hydrochloride (Sigma-Aldrich; # L9756) solution was prepared in water and dissolved in nematode growth medium to a concentration of 0.6 mm. Young adults animals (20–25) were placed on levamisole-containing plates seeded with OP50 and were monitored for paralysis after every 10 min. The animals were scored for paralysis by tapping the plates on the bench as has been previously described (Gendrel et al., 2009; Rapti et al., 2011). Animals showing less than one body bend were scored as paralyzed. The assay was performed in triplicates with the experimenter being blind to the genotype of the animals. For simplicity, the percentage of animals that were paralyzed at the 100 min time point was plotted.

##### Muscimol assay.

Muscimol (Sigma-Aldrich; # G019) assays were performed as described previously (de la Cruz et al., 2003). Briefly, 10–12 animals were placed on plates containing 200 mm muscimol for 1 h, after which the *C. elegans* behavior was analyzed by recording their response to touch. The animals were scored according to their movement or the pattern of contraction and relaxation cycle of the body. The behavior of *C. elegans* was classified into five categories: 0, *C. elegans* did not contract or relax but moved away from the stimulus rapidly; 1, *C. elegans* contract and relax briefly and then move away from the stimulus; 2, animals contracted and relaxed while showing a small backward displacement; 3, *C. elegans* contracted and relaxed but failed to move; and 4, animals contracted and relaxed incompletely with no displacement. The assay was performed in triplicates with the experimenter being blind to the genotype of the animals.

#### Microscopy

The imaging was done using a Axio Imager Z2 with an Axiocam MRm camera (Carl Zeiss). For imaging experiments, the animals were immobilized using 30 mg/ml BDM on 2% agarose pads. The analysis of images was done using the ImageJ software. For quantitative analysis, the fluorescence intensity of ∼25 animals (actual number indicated at the base of each bar graph) was averaged and used to plot the graph using the Prism software (GraphPad). Data are expressed as mean ± SEM. *p* values were based on Student's *t* test or one-way ANOVA. Images for muscle arms were acquired using the TCS SP8 confocal microscope (Leica), and the image processing was done using ImageJ software.

#### qPCR

The qPCR experiments were performed using total RNA extracted from mixed-stage animals using the RNA easy kit according to the manufacturer's instructions (QIAGEN). The RNA was transcribed to cDNA using the Transcriptor high-fidelity cDNA synthesis kit (Roche; # 05081955001). The qPCR was performed using SYBR Green master mix (QIAGEN; # 204141) using an Eppendorf thermal cycler.

#### Antibody production

The UNC-29 and UNC-38 antibodies were produced as previously described (Gally et al., 2004; Gendrel et al., 2009). Briefly, for the UNC-29 antibody, a DNA fragment encoding 348–431 amino acids of the UNC-29 gene was inserted into pGEX-5X and for the UNC-38 antibody, a DNA fragment encoding 375–418 amino acids of the UNC-38 gene was cloned in pGEX-5X. The GST::UNC-29 and GST::UNC-38 fusion proteins were then expressed in BL21 *Escherichia coli* cells and purified. This fusion protein was then injected into the rabbits, and rabbits were boosted with 100 μg each time. The antibody was then purified and used for experiments. The antibody production and purification were performed by Bioklone Biotech.

#### Immunoprecipitation and Western blotting

For protein extraction, mixed-stage animals were grown and stored at −80°C until further use. While starting the protein extraction, ice-cold homogenization buffer was added to the *C. elegans* pellets. *C. elegans* lysate was prepared by grinding the animals in a mortar and pestle in presence of liquid nitrogen. The suspension was centrifuged at 5000 × *g* for 10 min at 4°C to remove the debris. The supernatant was then centrifuged at high speed for 1 h at 4°C. The pellet was then dissolved in 100–500 μl of resuspension buffer as previously described (Gendrel et al., 2009). This fraction was then resolved on SDS-PAGE gel and blotted onto a nitrocellulose membrane. The membranes were then probed with the purified anti-UNC-29 (1:600) or anti-UNC-38 (1:1000) serum. Antirabbit secondary antibody (1:2000, IgG-AP # sc-2007, Santa Cruz Biotechnology) was used to probe the blot and detection was done using the Pierce Alkaline phosphatase substrate kit.

To perform the coimmunoprecipitation experiment, *C. elegans*-expressing HPO-30::mCherry in muscles was grown in multiple plates (40 plates of 90 mm diameter). The animals were collected using M9 buffer, and they were washed three times with the M9 buffer to get rid of the OP50. The protein was prepared using a previously described protocol (Gendrel et al., 2009). Next, 3 mg of protein sample was incubated with UNC-29 and UNC-38 antibodies at 4°C for 4 h; 50 μl of equilibrated protein A/G beads (Merck Millipore #16–125) was added to the lysate and further incubated for 3 h. Next, the beads were washed 5 times with ice-cold lysis buffer. The protein was then eluted in 30 μl of sample loading buffer by boiling for 5 min. Western blotting was performed using mCherry rat antibody (Molecular Probes # M11217) at 1:1000 dilution. We used HRP-labeled antirat secondary antibody at a dilution of 1:5000. Protein detection was done using the GE enhanced chemiluminescent detection reagent.

#### Electrophysiological recordings

Electrophysiology was done on dissected *C. elegans* as previously described (Hu et al., 2012; Liu et al., 2018). The *C. elegans* was superfused in an extracellular solution containing 127 mm NaCl, 5 mm KCl, 26 mm NaHCO_3_, 1.25 mm NaH_2_PO_4_, 20 mm glucose, 1 mm CaCl_2_, and 4 mm MgCl_2_, bubbled with 5% CO_2_/95% O_2_ at 20°C. Whole-cell recordings were performed at −60 mV (reversal potential of GABA_A_ receptors) for mEPSCs and 0 mV (reversal potential of AChRs) for mIPSCs. The internal solution contains 105 mm CH_3_O_3_SCs, 10 mm CsCl, 15 mm CsF, 4 mm MgCl_2_, 5 mm EGTA, 0.25 mm CaCl_2_, 10 mm HEPES, and 4 mm Na_2_ATP, adjusted to pH 7.2 using CsOH. Stimulus-evoked EPSCs were stimulated by placing a borosilicate pipette (51 μm) near the ventral nerve cord (one muscle distance from the recording pipette) and applying a 0.4 ms, 85 μA square pulse (WPI). To measure levamisole-activated currents, a puffing pipette (5–10 μm open size) containing 0.5 mm levamisole was placed at the end of the patched muscle, and a 100 ms 20 kPa pressure was applied via Picospritzer (Parker).

#### Statistical analysis

Statistical values for each set of genotypes compared through Student's *t* test are indicated as *p*, *t*, and *df*.

## Results

### Mutants of *hpo-30*, a claudin-like protein, are resistant to aldicarb

To understand the function of claudins at the NMJ, we screened through all available claudin mutants using the acetylcholine esterase inhibitor, aldicarb. Aldicarb causes an increase in the levels of acetylcholine and hence results in increased muscle contraction in the animal, which in turn leads to paralysis. Mutants with altered synaptic function show increased or decreased rates of paralysis compared with WT *C. elegans* (Miller et al., 1996; Sieburth et al., 2005; Vashlishan et al., 2008).

**Figure 1.**
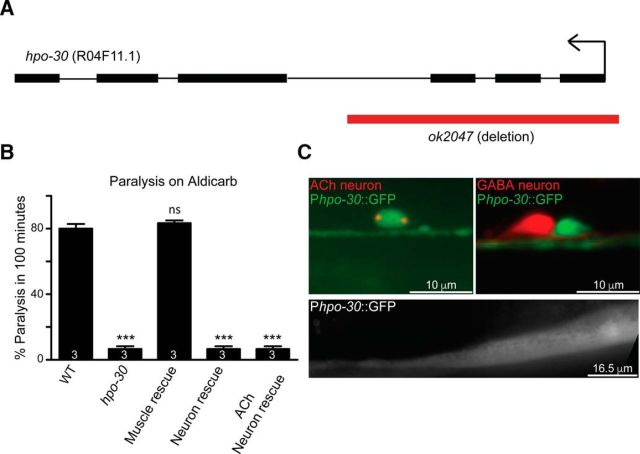
Mutants in *hpo-30* are resistant to aldicarb. ***A***, Schematic of the *hpo-30* gene. Black boxes represent the coding exons. Red bar represents the *ok2047* deletion. ***B***, Aldicarb assay of WT, *hpo-30* mutants, and site-specific rescue of HPO-30. Bar graphs represent the percentage of animals paralyzed at the 100 min time point. In all the aldicarb graphs, the number at the base of the bars indicates the number of times the assay was performed with 20–25 *C. elegans* used for each trial. Data are mean ± SEM. ***p < 0.001. ***C***, Transcriptional reporter of *hpo-30* shows expression in cholinergic neurons and not in GABAergic neurons, which were identified by P*unc-17*::mCherry and P*unc-25*::mCherry, respectively. P*hpo-30*::GFP also shows expression in body-wall muscles. ns, not significant.

In this screen, we found that a deletion (likely null) mutant of *hpo-30* (*ok2047*) that removes 1.2 kb of the coding region, including exons 1–3 showed an extremely resistant phenotype upon exposure to aldicarb compared with WT animals (WT and *hpo-30*, *p* = 0.0002, *t* = 22, df = 4; Fig. 1*A*,*B*). HPO-30 encodes a four-transmembrane domain protein with similarity to members of the claudin-like family of proteins.

Previous studies have shown the expression of HPO-30 in FLP, PVD, tail, and ventral cord neurons and that it is required for stabilizing dendritic branching in PVD neurons (Smith et al., 2013). Because we were interested in understanding the role of HPO-30 at the NMJ, we planned to evaluate the expression of the *hpo-30* promoter in motor neurons and/or body-wall muscles. To achieve this, we made a GFP transcriptional reporter containing a 3 kb upstream region of *hpo-30* and analyzed the expression of this promoter fusion. We observed the expression of HPO-30 in the ventral cord motor neurons as previously reported, further diffuse expression was present in the body-wall muscles (data not shown; and Fig. 1*C*) (Smith et al., 2013). To identify the HPO-30-expressing neurons, we performed a double-labeling experiment and found that the GFP-tagged *hpo-30* promoter showed coexpression with mCherry-labeled cholinergic neurons and not with GABAergic neurons (Fig. 1*C*).

Because the *hpo-30* promoter showed expression in cholinergic neurons and body-wall muscle, we next went on to find the site of action of HPO-30. To achieve this, we expressed HPO-30 specifically in the body-wall muscles, pan-neuronally and in cholinergic neurons. The data from the rescue experiments indicated that the aldicarb resistance in *hpo-30* mutants could be rescued only when HPO-30 was expressed specifically in the body-wall muscles (WT and *hpo-30*; P*myo-3*::HPO-30, *p* = 0.3910, *t* = 1, df = 3; Fig. 1*B*).

Because HPO-30 showed expression in presynaptic cholinergic neurons and postsynaptic body-wall muscles, we next wanted to see whether the mutants showed defects in synaptic development.

### Synapse morphology is normal in *hpo-30* mutants

**Figure 2.**
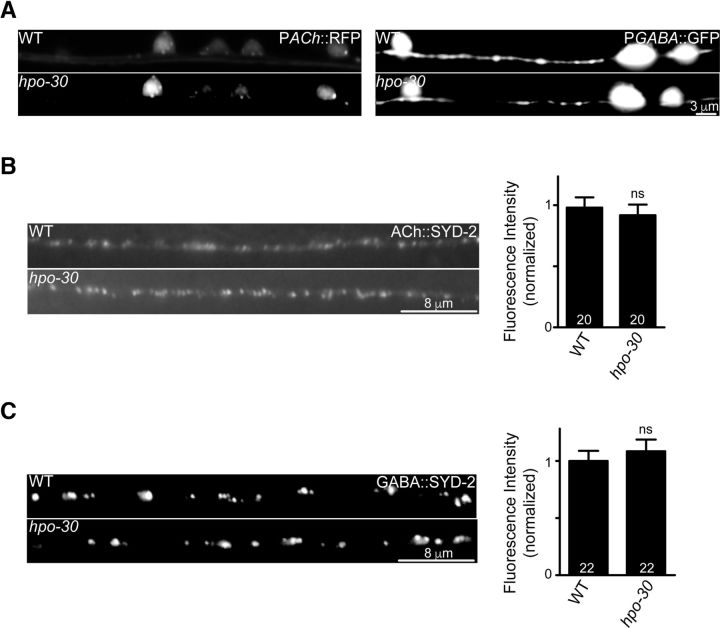
*hpo-30* mutants have normal neuromuscular synapses. ***A***, Expression of P*unc-17*::mCherry and P*unc-25*::GFP in WT and *hpo-30* mutant animals. ***B***, Representative images and quantification for the active zone protein SYD-2 along the dorsal cord axons of cholinergic neurons in WT and *hpo-30 C. elegans*. ***C***, Representative images and quantification for active zone protein SYD-2 in dorsal cord axons of GABAergic neurons in WT and *hpo-30* animals. Data are mean ± SEM. ns, not significant.

The decreased responsiveness of *hpo-30* mutant animals toward aldicarb could be due to altered neuronal or synapse development. To check for developmental defects in neurons and synapses, we went on to look at a set of neuronal and synaptic markers in the *hpo-30* mutant animals. Loss of *hpo-30* did not appear to have any obvious developmental defects on the cholinergic or GABAergic motor neurons (Fig. 2*A*). Further, both cholinergic and GABAergic synapses at the NMJ appeared to be formed normally as seen using the active zone marker α-liprin/SYD-2 expressed specifically in cholinergic or GABAergic neurons, respectively (WT and *hpo-30*, *p* = 0.6081, *t* = 0.5168, df = 41; Fig. 2*B*; and WT and *hpo-30*, *p* = 0.5299, *t* = 0.6331, df = 41; Fig. 2*C*). Together, these results suggest that the decreased rate of paralysis shown by *hpo-30* mutant animals is unlikely to be due to developmental defects of neurons or synapses.

### *hpo-30* mutants have normal synaptic vesicle release at the NMJ

**Figure 3.**
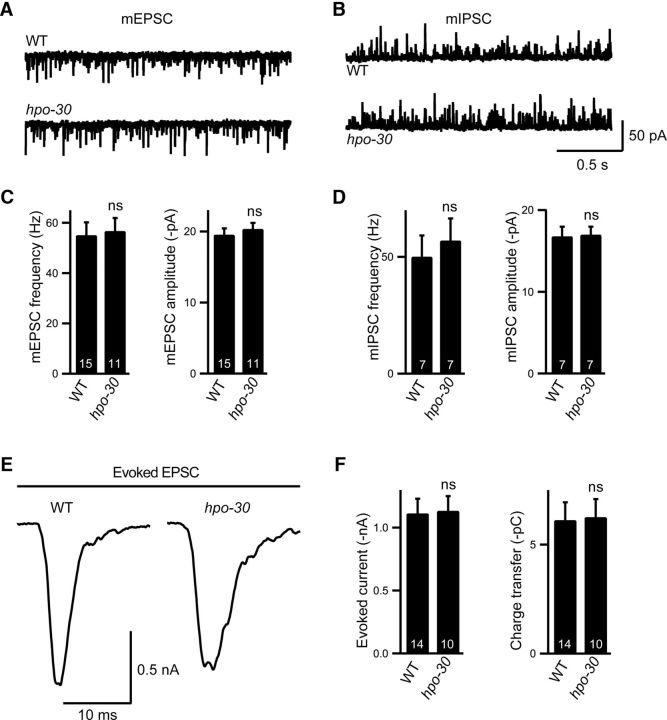
Endogenous and evoked neurotransmitter release is normal in *hpo-30* mutants. ***A***, Representative trace of endogenous EPSC recordings from body-wall muscles of adult WT and *hpo-30* animals. ***B***, Representative trace of endogenous IPSC recordings from body-wall muscle of adult WT and *hpo-30* mutants. ***C***, Average frequency and average amplitude of EPSCs of WT and *hpo-30* mutants. The numbers at the base of the bars indicate the number of animals recorded in all figures. ***D***, Average frequency and average amplitude of IPSCs for WT and *hpo-30* mutant *C. elegans*. ***E***, Trace of stimulus-evoked response measured from the body-wall muscles of adult animals. ***F***, Summary data of evoked currents in WT and *hpo-30* mutants. Data are mean ± SEM.

The aldicarb resistance phenotype showed by *hpo-30* mutants and the expression of P*hpo-30*::GFP in cholinergic neurons and body-wall muscles indicated a role for HPO-30 in either promoting the vesicle release machinery or maintaining the acetylcholine reception machinery. To assay synaptic transmission, we measured EPSCs and IPSCs from the body-wall muscles (Richmond, 2006; Hu et al., 2012). The amplitude and rate of endogenous EPSCs and IPSCs were unaltered in *hpo-30* mutants (Fig. 3*A–D*: frequency WT and *hpo-30*, *p* = 0.8281, *t* = 0.2195, df = 24; amplitude, *p* = 0.5499, *t* = 0.6065, df = 24; Fig. 3*C*; WT and *hpo-30* frequency, *p* = 0.6177, *t* = 0.5123, df = 12; and amplitude, *p* = 0.8981, *t* = 0.1308, df = 12; Fig. 3*D*). This suggests that synaptic transmission at the cholinergic and GABAergic NMJs is largely normal. Further, we looked at the muscle responsiveness by measuring the evoked currents and found that there were no significant changes in the amplitude and charge transfer of evoked EPSCs in the *hpo-30* animals compared with WT controls (Fig. 3*E*: evoked EPSC amplitude WT and *hpo-30*, *p* = 0.9087, *t* = 0.1160, df = 22; and charge, *p* = 0.8852, *t* = 0.1461, df = 22; Fig. 3*F*). The *C. elegans* body-wall muscle possesses two types of cholinergic receptors: ACR-16 and LAChRs (Lewis et al., 1980; Fleming et al., 1997; Richmond and Jorgensen, 1999; Culetto et al., 2004; Touroutine et al., 2005; Towers et al., 2005). ACR-16 receptors account for ∼90% of the postsynaptic currents in *C. elegans*, and changes in ACR-16 levels affect the amplitude of the EPSCs and cause changes in the evoked EPSC current amplitudes (Francis et al., 2005; Touroutine et al., 2005; Babu et al., 2011). Hence, these data showing no significant changes in evoked EPSC amplitude suggest that HPO-30 does not appear to affect ACR-16 receptors. We next went on to further test whether HPO-30 is required for normal postsynaptic receptor function.

### HPO-30 is required for normal LAChR-dependent functions

The decreased rate of paralysis of *hpo-30* mutants in the presence of aldicarb and the ability to rescue this defect by expressing HPO-30 specifically in body-wall muscles suggest that HPO-30 could function in the body-wall muscles for maintaining normal aldicarb response.

The *C. elegans* NMJ expresses receptors that respond to both cholinergic and GABAergic neurotransmitters functioning at the body-wall muscles (Richmond and Jorgensen, 1999). To explore the possibility that HPO-30 could affect receptor function at the body-wall muscles and thereby show altered aldicarb sensitivity, we performed pharmacological experiments to assay for defects in cholinergic and GABAergic receptors.

**Figure 4.**
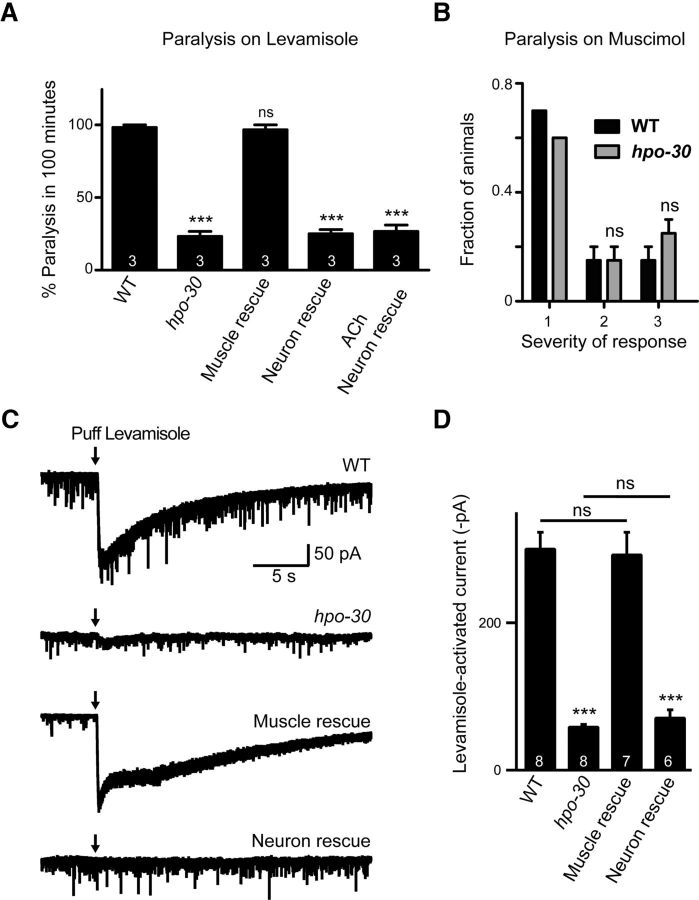
*hpo-30* mutants show defects in LAChR functions. ***A***, Levamisole-induced paralysis of WT, *hpo-30* mutants, and site-specific rescue of HPO-30. Graph represents percentage of paralyzed animals at the 100 min time point. ***B***, Behavior of WT and *hpo-30* mutant animals in the presence of muscimol. Graph represents the fraction of *C. elegans*, which displayed the three levels of severity of behavior in the presence of muscimol. The behavior of the animals was classified onto five categories, of which both WT and *hpo-30* animals showed only three of the phenotypes: 1, *C. elegans* contract and relax briefly and then move away from the stimulus; 2, animals contract and relax while at the showing a small backward displacement; and 3, *C. elegans* contract and relax but fail to move backwards. ***C***, Response to pressure ejection of levamisole on voltage-clamped body-wall muscle cells in animals with the following genotypes: WT, *hpo-30*, and muscle-specific rescue and neuron-specific rescue of HPO-30. ***D***, Graph represents the amplitude of levamisole-activated currents for the animals recorded in ***C***. Data are mean ± SEM. ***p < 0.001.

As stated previously, the cholinergic receptors include two classes of nicotinic AChRs: the homomeric AChR/ACR-16 receptors and the heteropentameric LAChRs, which are activated specifically by the drug levamisole (Lewis et al., 1980; Fleming et al., 1997; Richmond and Jorgensen, 1999; Culetto et al., 2004; Touroutine et al., 2005; Towers et al., 2005). Levamisole is an agonist of the AChR and causes muscle hypercontraction, leading to the paralysis of the animals. Mutants lacking the ACR-16 receptors have a mild change in aldicarb sensitivity, whereas mutants lacking the LAChRs exhibit strong resistance to aldicarb (Lewis et al., 1980; Daniels et al., 2000; Babu et al., 2011), suggesting that HPO-30 may regulate the functioning of LAChRs. To see whether *hpo-30* mutants affected LAChRs, we performed a levamisole assay using the *hpo-30* mutant animals (Lewis et al., 1980; Gally et al., 2004). Mutants in *hpo-30* showed resistance to paralysis in the presence of levamisole (WT and *hpo-30*, *p* < 0.0001, *t* = 20.12, df = 4); this resistance was rescued by the expression of HPO-30 specifically in the body-wall muscles and not in motor neurons (WT and *hpo-30*; P*myo-3*::HPO-30, *p* = 0.6779, *t* = 0.4472, df = 4; Fig. 4*A*). This experiment indicated that HPO-30 could be affecting LAChRs at the body-wall muscle. We also performed assays for defects in the GABA receptor at the NMJ using the drug muscimol, which is a GABA receptor agonist (de la Cruz et al., 2003). In the presence of muscimol, we saw no difference between WT and *hpo-30* mutant animals, indicating that loss of *hpo-30* does not appear to affect GABA receptors (Severity 2, *p* = 1.00, *t* = 0.0, df = 2; Severity 3, *p* = 0.2929, *t* = 1.414, df = 2; Fig. 4*B*).

Because all our data so far indicate that *hpo-30* does not appear to affect either the amplitude of the EPSCs or the evoked currents, we hence reasoned that HPO-30 was unlikely to be affecting ACR-16 levels at the NMJ (Fig. 3*A*,*C*,*E*,*F*).

To better understand the functional consequence of this decreased sensitivity toward levamisole, we recorded the levamisole puff response of body-wall muscles in *hpo-30* mutant animals using previously described protocols (Richmond, 2006; Hu et al., 2012). We observed a large decrease in the amplitude of the currents in *hpo-30* mutants compared with WT animals (WT and *hpo-30*, *p* < 0.0001, *t* = 29.37, df = 14). Again, this decrease in levamisole puff response was rescued by expressing HPO-30 in the body-wall muscles (WT and *hpo-30*; P*myo-3*::HPO-30, *p* = 0.5674, *t* = 0.5868, df = 13; Fig. 4*C*,*D*), whereas HPO-30 expression pan-neuronally could not rescue this phenotype (WT and *hpo-30*; P*rab-3*::HPO-30, *p* < 0.0001, *t* = 27.58, df = 12; Fig. 4*C*,*D*). The above data strongly suggest that HPO-30 functions in the body-wall muscles to maintain the function of LAChRs. These results prompted us to look at LAChR levels at the NMJ.

### HPO-30 is involved in the maintenance of LAChR levels at the NMJ

**Figure 5.**
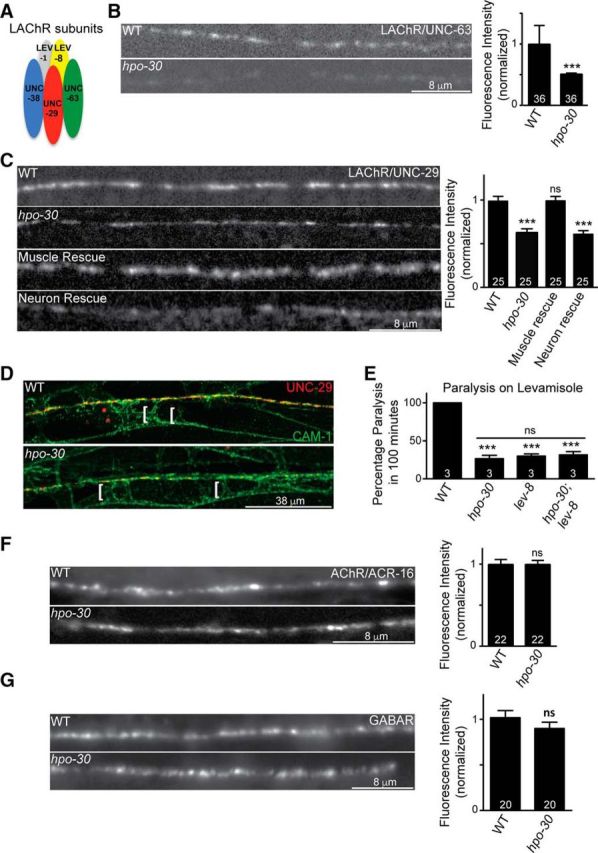
Mutants in *hpo-30* show a decrease in synaptic LAChR abundance. ***A***, Diagram of five distinct subunits of the LAChRs. ***B***, Distribution of UNC-63::YFP at the NMJ in WT and *hpo-30* animals and the fluorescence intensity of UNC-63::YFP in WT and *hpo-30*. The number of animals analyzed is indicated at the base of the bar graph for all graphs. ***C***, Representative images and quantification for distribution of UNC-29::GFP at the NMJ in WT, *hpo-30*, and HPO-30 rescue lines. ***D***, Localization of UNC-29::RFP and CAM-1::GFP at the NMJ in WT and *hpo-30* animals. Brackets indicate the muscle arms. ***E***, Levamisole-induced paralysis in WT, *hpo-30*, *lev-8*, and *hpo-30;lev-8* mutants at the 100 min time point. ***F***, Representative images and quantification for the distribution of ACR-16::GFP at the NMJ in WT and *hpo-30* animals. ***G***, Representative images and quantification for the distribution of UNC-49::GFP at the NMJ in WT and *hpo-30* mutants. Data are mean ± SEM. ***p < 0.001.

Decreased sensitivity of *hpo-30* mutants to levamisole could be the result of altered expression or function of LAChRs. The LAChRs are pentameric receptors composed of five subunits: UNC-29, UNC-63, UNC-38, LEV-8, and LEV-1 (Fig. 5*A*) (Fleming et al., 1997; Richmond and Jorgensen, 1999; Culetto et al., 2004; Towers et al., 2005). We first went on to look at the expression of fluorescently tagged LAChR/UNC-29 and LAChR/UNC-63 receptor subunits in the *hpo-30* mutant animals (Francis et al., 2005; Rapti et al., 2011). We found that *hpo-30* mutants showed a significant decrease in the fluorescence intensity of both UNC-63::YFP and UNC-29::GFP at the NMJ (WT and *hpo-30*, *p* = 0.0004, *t* = 3.450, df = 66; Fig. 5*B*; WT and *hpo-30*, *p* < 0.0001, *t* = 5.973, df = 43; Fig. 5*C*). The decreased levels of UNC-29::GFP were completely rescued by specifically expressing HPO-30 in the body-wall muscles (WT and *hpo-30*; P*myo-3*::HPO-30, *p* = 0.9668, *t* = 0.0418, df = 44) but not in the neurons of the animals (WT and *hpo-30*; P*rab-3*::HPO-30, *p* = 0.0001, *t* = 6.234, df = 40; Fig. 5*C*).

At the *C. elegans* NMJ, body-wall muscles extend projections called muscle arms to the nerve cord where they form *en passant* synapses with axons of motor neurons (White et al., 1986). The decrease in the fluorescence of LAChR/UNC-29 at the neuromuscular synapse in *hpo-30* mutant animals could be because the receptors are not localized at the synapse but are localized extrasynaptically in the regions of the muscle arms. To test whether LAChR/UNC-29 was localized to the body-wall muscle arms in the mutants, we studied the localization of LAChR/UNC-29 at extrasynaptic regions using a strain in which the fluorescent protein tagRFP is introduced into the genomic locus of UNC-29 (Robert and Bessereau, 2007; Richard et al., 2013). We found that, in this line as well, there was a marked decrease in synaptic LAChR/UNC-29 in *hpo-30* mutants (representative image in Fig. 5*D*), but no mislocalization of the receptors in the region of the body-wall muscle arms, indicated with CAM-1::GFP, was seen (Fig. 5*D*, white brackets). Together, these results suggest that loss of *hpo-30* decreases the synaptic abundance of LAChRs, and it is unlikely that the decrease in the LAChR levels is due to the mislocalization of the receptors at the site of muscle arms.

To further understand the function of HPO-30 in maintaining LAChRs at the synapse, we analyzed the behavior of *hpo-30* mutants, *lev-8* mutants, and *hpo-30;lev-8* double mutants in the presence of levamisole. The resistance phenotypes of both single mutants and the double mutant were indistinguishable (*hpo-30* and *hpo-30;lev-8*, *p* = 0.4674, *t* = 0.8018, df = 4; Fig. 5*E*), thus suggesting that another subunit of LAChR functions in the same pathway as HPO-30.

Next, we wanted to test whether HPO-30 affected other receptors; we assayed for defects in the fluorescently tagged homomeric AChR/ACR-16 and the GABA_A_R/UNC-49 (Bamber et al., 1999; Babu et al., 2011). It was found that the levels of ACR-16::GFP and UNC-49::GFP were not affected by the absence of *hpo-30* (WT and *hpo-30*, *p* = 1.0000, *t* = 0.000, df = 42; Fig. 5*F*; and WT and *hpo-30*, *p* = 0.2650, *t* = 1.135, df = 32; Fig. 5*G*).

These results suggest that HPO-30 function in muscles to maintain normal LAChR levels at the *C. elegans* NMJ. We next wanted to test whether HPO-30 affected the expression levels of LAChRs.

### Mutants of *hpo-30* show normal expression of LAChR subunits

**Figure 6.**
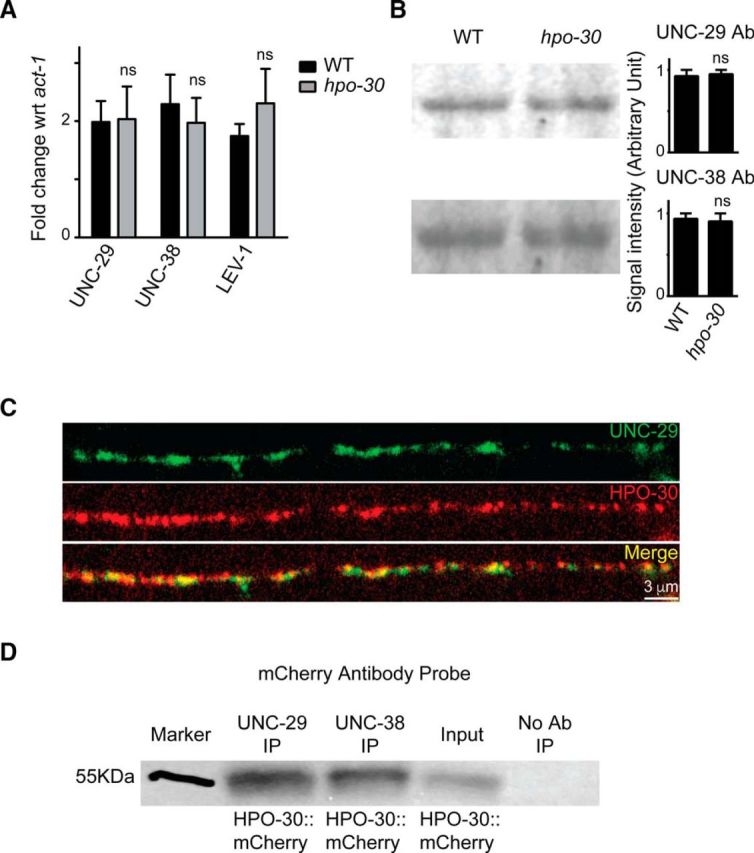
The expression level of LAChRs is unaffected in *hpo-30* mutants. ***A***, qPCR experiments comparing the levels of *unc-29*, *unc-38*, and *unc-63* in WT and *hpo-30* mutant animals. ***B***, Images and graphs of Western blots indicating the protein levels of UNC-29 and UNC-38 in WT and *hpo-30 C. elegans*. Data are mean ± SEM. ***C***, Distribution of HPO-30::mCherry and UNC-29::GFP in muscles. ***D***, Coimmunoprecipitation of HPO-30::mCherry using anti-UNC-29 and anti-UNC-38 antibodies. As a control, the protein extracts are allowed to incubate with beads only without the antibody. The coimmunoprecipitated complex was then probed with an anti-mCherry antibody.

Our experiments so far suggest that *hpo-30* mutant animals show a decrease in LAChR levels at the NMJ. The decrease in the levels of LAChRs could be the result of decrease in the expression of genes coding for LAChR subunits. To check whether HPO-30 is required for gene expression, we compared the expression levels of LAChR subunits *unc-29*, *unc-63*, and *unc-38* in WT and *hpo-30* mutant background by performing qRT-PCR. There was no apparent difference in the transcript levels of the three subunits of LAChR tested in WT and *hpo-30* mutants (*unc-29*, *p* = 0.9526, *t* = 0.06717, df = 2; *unc-38*, *p* = 0.6726, *t* = 0.4900, df = 2; and *lev-1*, *p* = 0.4612, *t* = 0.9046, df = 2; Fig. 6*A*). We also went on to quantitate the total amounts of UNC-29 and UNC-38 proteins in the animals by quantitative Western blotting using antibodies against UNC-29 and UNC-38, respectively. Again, we found that there was no difference between the UNC-29 and UNC-38 protein levels in WT and *hpo-30* mutant animals (UNC-29, *p* = 0.8076, *t* = 0.2773, df = 2; and UNC-38, *p* = 0.8188, *t* = 0.2606, df = 2; Fig. 6*B*). These results indicate that HPO-30 is unlikely to be required for the transcription or the translation of the LAChR subunits. To further analyze the association between HPO-30 and LAChRs, we analyzed the localization of mCherry tagged HPO-30 expressed in body-wall muscles and observed overlapping expression with LAChRs/UNC-29 postsynaptically at the NMJ (Fig. 6*C*).

As HPO-30 showed genetic interaction with the LAChR subunit LEV-8 and also colocalized with UNC-29 (Figs. 5*E*, 6*C*), we hypothesized that HPO-30 might be functioning through a physical interaction where it may complex with LAChR subunits. To test this, we performed a coimmunoprecipitation experiment using *C. elegans* expressing HPO-30::mCherry in muscles. We used anti-UNC-29 and anti-UNC-38 to find the probable interaction between these LAChRs and HPO-30. Upon blotting with an antibody against mCherry, we found that HPO-30::mCherry was pulled down with UNC-29 and UNC-38 antibodies. As a control, we used protein lysate incubated with beads only and no antibody (Fig. 6*D*). These results demonstrate that HPO-30 shows a possible association with the LAChRs UNC-29 and UNC-38 and shows colocalization with UNC-29 at the NMJ. Together, these data indicate that HPO-30 may be required for stabilizing the LAChRs at the NMJ.

### HPO-30 functions through Neuroligin to maintain normal levamisole puff response

**Figure 7.**
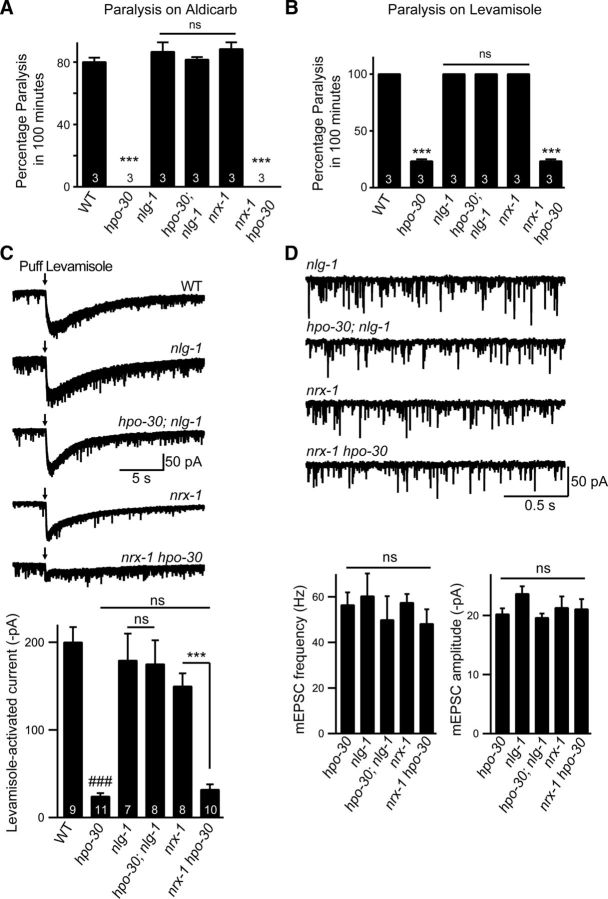
HPO-30 functions through NLG-1 to maintain LAChR function. ***A***, Percentage paralysis of animals at the 100 min time point in WT (80%), *hpo-30* (0%), *nlg-1* (90%), *nrx-1* (90%), *hpo-30;nlg-1* (80%), and *nrx-1 hpo-30* (0%) in the presence of aldicarb. ***B***, Percentage paralysis of animals at 100 min in WT (100%), *hpo-30* (25%), *nlg-1* (100%), *nrx-1* (100%), *hpo-30;nlg-1* (100%), and *nrx-1 hpo-30* (25%) upon exposure to levamisole. ***C***, Currents recorded from voltage-clamped body-wall muscles in response to pressure-ejected levamisole in WT, *nlg-1*, *hpo-30;nlg-1*, *nrx-1*, and *nrx-1 hpo-30*. Black arrow indicates the time of application of levamisole and summary data for levamisole-puff currents. ***D***, Representation of endogenous EPSCs recorded from body-wall muscles of adult *C. elegans* for the following genotypes: *nlg-1*, *hpo-30;nlg-1*, *nrx-1*, and *nrx-1;hpo-30* and graphs showing average frequency and amplitude of EPSCs recorded. Data are mean ± SEM. ***p < 0.001, ^###^*p* < 0.001.

The body-wall muscles in *C. elegans* cluster GABAergic receptors apposing GABAergic inputs and LAChRs and ACR-16 receptors apposing cholinergic inputs (White et al., 1976, 1986; Richmond and Jorgensen, 1999). Recent studies have shown that Neurexin (NRX-1) and NLG-1 are required for clustering GABA_A_ receptors at the NMJ (Maro et al., 2015; Tu et al., 2015). To test whether HPO-30 could function through NRX-1 and/or NLG-1, we analyzed the behavioral responses of *hpo-30;nlg-1* and *nrx-1;hpo-30* double mutants toward aldicarb and levamisole along with their respective controls (i.e., single mutants for *hpo-30*, *nlg-1*, and *nrx-1*). Surprisingly, we found that the *hpo-30;nlg-1* mutants, but not the *nrx-1;hpo-30* mutants, could completely suppress the resistance to aldicarb and levamisole phenotype that was seen in the *hpo-30* mutants (aldicarb assay; WT and *hpo-30;nlg-1*, *p* = 0.6433, *t* = 0.50, df = 4; Fig. 7*A*). However, *nlg-1* and *nrx-1* single mutants showed phenotypes similar to WT animals on aldicarb and levamisole (WT and *nlg-1*, *p* = 0.3739, *t* = 1.000, df = 4; and WT and *nrx-1*, *p* = 0.1890, *t* = 1.581, df = 4). RNAi knockdown of *nlg-1* and *hpo-30* also confirmed our experiments performed with genetic mutants (data not shown).

To further understand the role of NLG-1 in maintaining postsynaptic LAChRs, we performed levamisole-puff response-based electrophysiological analysis on the *hpo-30;nlg-1* and the *nrx-1;hpo-30* double mutants. Again, we found that *nlg-1* and not *nrx-1* could completely suppress the loss of levamisole puff response of *hpo-30* mutants (WT and *hpo-30*, *p* < 0.0001, *t* = 11.32, df = 18; WT and *nlg-1*, *p* = 0.5356, *t* = 0.6350, df = 14; WT and *nrx-1*, *p* = 0.0403, *t* = 2.244, df = 15; WT and *hpo-30;nlg-1*, *p* = 0.4315, *t* = 0.8084, df = 15; WT and *nrx-1 hpo-30*, *p* < 0.0001, *t* = 9.911, df = 17; Fig. 7*C*).

We also recorded the endogenous EPSCs from the muscles in *hpo-30;nlg-1* and *nrx-1 hpo-30* mutant lines along with the single mutant controls and found that both the frequency and amplitude of the postsynaptic current were not significantly different from the single mutants (EPSC frequency; *hpo-30* and *nlg-1*, *p* = 0.4122, *t* = 0.8419, df = 16; *hpo-30* and *hpo-30;nlg-1*, *p* = 0.5368, *t* = 0.6322, df = 15; EPSC amplitude; *hpo-30* and *nlg-1*, *p* = 0.0328, *t* = 2.336, df = 16; *hpo-30* and *hpo-30;nlg-1*, *p* = 0.6583, *t* = 0.4513, df = 15; Fig. 7*D*). Together, these results suggest that NLG-1 and NRX-1 are unlikely to be required for the functioning of ACR-16 receptors at the NMJ.

Our results indicate that HPO-30 could function through NLG-1 to maintain the LAChR-dependent functions at the NMJ.

### NLG-1 functions downstream of HPO-30

**Figure 8.**
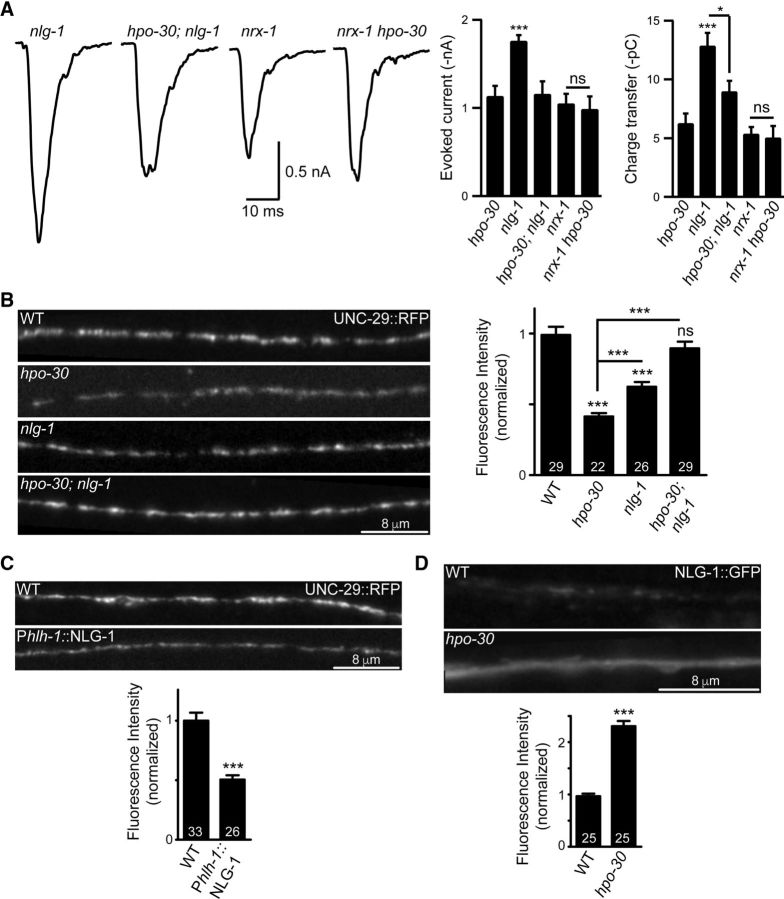
NLG-1 functions downstream of HPO-30. ***A***, Stimulus-evoked responses measured from the body-wall muscles of *nlg-1*, *hpo-30;nlg-1*, *nrx-1*, and *nrx-1 hpo-30* animals and summary data for amplitude of evoked currents and charge transfer. ***B***, Representative images and quantification for UNC-29::tagRFP in WT, *hpo-30*, *nlg-1*, and *hpo-30;nlg-1*. ***C***, Representative images and quantification for UNC-29::tagRFP in WT and NLG-1-overexpressing animals. ***D***, Representative images and quantification for NLG-1::GFP in WT and *hpo-30* mutant *C. elegans*. Data are mean ± SEM. *p < 0.05, ***p < 0.001.

Our data so far indicate that loss of *nlg-1* could suppress the *hpo-30* mutant phenotype. We also analyzed the stimulus-evoked muscle responsiveness in *nlg-, hpo-30*, and *hpo-30;nlg-1* mutants. We found that the stimulus-evoked currents in the double mutants were also similar to the control values (evoked currents; *hpo-30* and *nlg-1*, *p* = 0.0007, *t* = 4.203, df = 16; and *hpo-30* and *hpo-30;nlg-1*, *p* = 0.9020, *t* = 0.1253, df = 14; charge transfer; *hpo-30* and *nlg-1*, *p* = 0.0002, *t* = 4.761, df = 16; and *hpo-30* and *hpo-30;nlg-1*, *p* = 0.0590, *t* = 2.055, df = 14; Fig. 8*A*).

As all the previous results indicate that the absence of *nlg-1* and *hpo-30* could affect LAChR levels at the NMJ, we went on to examine the LAChR/UNC-29 levels in the *hpo-30;nlg-1* double mutants. We analyzed the expression of UNC-29::tagRFP in *hpo-30*, *nlg-1*, and *hpo-30;nlg-1* genetic backgrounds. We observed a significant decrease in the florescence intensity of UNC-29 at the NMJ in the *hpo-30* mutant background (WT and *hpo-30*, *p* < 0.0001, *t* = 8.283, df = 49; Fig. 8*B*). However, in the *hpo-30;nlg-1* double mutants, the florescence intensity was comparable with WT animals (WT and *hpo-30;nlg-1*, *p* = 0.2109, *t* = 1.269, df = 56; Fig. 8*B*). These results further indicate that *nlg-1* suppresses the *hpo-30* mutant phenotype to WT levels of LAChR/UNC-29 at the NMJ. Surprisingly, we also see a significant decrease in the LAChR/UNC-29 receptor levels in *nlg-1* mutants (WT and *nlg-1*, *p* < 0.0001, *t* = 5.3089, df = 53; Fig. 8*B*), which we are unable to explain, as there is no significant change in the levamisole puff response in these animals (Fig. 7*C*). This change in the UNC-29 receptor levels at the NMJ in the absence of *nlg-1* may indicate that NLG-1 could have a possible function in maintaining the LAChRs at the NMJ.

Our data suggest that elevated NLG-1 levels could result in decreased LAChRs at the NMJ. To verify this hypothesis, we looked at the expression of LAChR/UNC-29 after overexpressing NLG-1 in muscles. We found that there is a marked reduction in the UNC-29::tagRFP levels at the NMJ compared with WT control animals (*p* < 0.0001, *t* = 5.818, df = 57; Fig. 8*C*).

Finally, we wanted to test whether HPO-30 is required for maintaining NLG-1 levels at the NMJ. To do this, we imaged NLG-1::GFP in WT animals and in *hpo-30* mutants. We found a significant increase in NLG-1 at the NMJ in *hpo-30* mutants (*p* < 0.0001, *t* = 12.55, df = 46; Fig. 8*D*). These experiments further strengthened our hypothesis that NLG-1 could function downstream of HPO-30. Expression of HPO-30 is required to maintain WT levels of NLG-1 at the synapse, and loss of *hpo-30* shows an aberrant increase in the synaptic NLG-1 levels. Together, our data strongly suggest that HPO-30 maintains LAChR levels at the NMJ and is functioning through NLG-1 to maintain its function at the neuromuscular synapse.

## Discussion

Neurotransmission occurs at specialized points of contacts between the presynaptic neuron and the postsynaptic neuron or muscle. The signaling at the NMJ demonstrates a high level of subcellular complexity, and we are still to get a clear understanding of the NMJ components and their signaling mechanisms. In this study, we set out to determine the function of a *C. elegans* claudin-like molecule HPO-30 at the NMJ. Our results indicate that the expression and function of HPO-30 in the body-wall muscles are required to maintain normal LAChR receptor levels at the NMJ. Mutants lacking this protein exhibit resistance to cholinergic but not GABAergic agonists, indicating that HPO-30 affects AChRs and not GABARs.

In *C. elegans*, two types of AChRs are present at the NMJ. Pharmacologically, one is preferentially gated by levamisole (LAChRs) and the other by nicotine (nAChR). The kinetics and the mechanism of regulation and clustering of these receptors are very different (Richmond and Jorgensen, 1999; Francis et al., 2005; Boulin et al., 2008, 2012; Simon et al., 2008; Gendrel et al., 2009; Babu et al., 2011; Rapti et al., 2011; Jensen et al., 2012; Richard et al., 2013; Briseño-Roa and Bessereau, 2014; Pinan-Lucarré et al., 2014; Pierron et al., 2016; Pandey et al., 2017). Because loss of LAChRs causes resistance of the animal to levamisole, genetic screens based on levamisole-induced paralysis have contributed to the identification of proteins involved in functioning or assembly of LAChRs (Lewis et al., 1980; Fleming et al., 1997).

Previously, claudin-like proteins have been implicated in regulating structural aspect of the *C. elegans* nervous system. NSY-4 has been shown to affect AWC neuron specification by regulating calcium channels (Vanhoven et al., 2006), whereas HPO-30 has been implicated in governing dendritic branching in PVD neurons (Smith et al., 2013).

Our studies highlight the role of HPO-30 for the proper localization and hence activity of LAChRs while leaving the nAChR/ACR-16 physiology unaffected. Our data also indicate a possible association between HPO-30 and LAChRs as we show that HPO-30 coimmunoprecipitates with LAChR subunits UNC-38 and UNC-29. Together, these results suggest that HPO-30 could have a role in the trafficking, assembly, stability, or clustering of the levamisole sensitive receptors at the *C. elegans* NMJ. Further experiments would be required to pinpoint the exact function of HPO-30 in LAChR maintenance.

Our results add to the growing body of work on the role of claudins in the nervous system, where we show that a claudin-like molecule acts at the neuromuscular synapse to maintain normal LAChR localization and function at the *C. elegans* NMJ.

The biogenesis and assembly of ionotropic receptors are a multistep process. Multiple proteins are involved in the folding, generation of membrane topology, and assembly of subunits into pentamers, trafficking and clustering of AChRs. The ligand-gated ion channels require auxiliary subunits for their trafficking, assembly, and function (for review, see Green, 1999; Keller and Taylor, 1999). Auxiliary proteins required for functioning and assembly of LAChRs in *C. elegans* include RIC-3, UNC-50, UNC-74, NRA-2, NRA-4, and RSU-1. RIC-3 and UNC-50 are required for the efficient assembly, maturation, and trafficking of receptors from ER (Halevi et al., 2002; Eimer et al., 2007; Boulin et al., 2008; Jospin et al., 2009). Recently, NRA-2/Nicalin (Nicastrin-like protein), NRA-4/nodal modulator, and RSU-1 are proposed to regulate LAChRs subunit composition, stoichiometry, and distribution (Almedom et al., 2009; Pierron et al., 2016). LEV-9, LEV-10, and OIG-4 serve as an extracellular scaffold that forms a physical complex with the LAChRs and localize them at the synapse (Gendrel et al., 2009; Rapti et al., 2011; Briseño-Roa and Bessereau, 2014). Our work on HPO-30 might help in understanding the mechanism that regulates receptor clustering or localization at the synapse. In the *C. elegans* nervous system, acetylcholine serves as the main excitatory neurotransmitter, the presence of multiple auxiliary proteins might represent a means to increase the functional repertoire of ionotropic AChRs. It will be interesting to understand how HPO-30 functions with other modulators in selectively regulating levamisole-sensitive ion channels.

Finally, we have identified NLG-1 as an important molecule in maintaining LAChRs through HPO-30 at the NMJ. Mutants in *nlg-1* have been previously shown to have defects in GABA_A_ receptor clustering and for normal retrograde signaling across the NMJ in *C. elegans* (Hu et al., 2012; Maro et al., 2015; Tu et al., 2015; Tong et al., 2017). A previous study by Hu et al. (2012) has shown that *nrx-1* and *nlg-1* inhibit the retrograde signal induced by the microRNA *mir-1* and that this inhibition is mediated by the presynaptic protein Tomosyn. Our data indicate that, unlike the other two components involved in retrograde signaling (i.e., *nlg-1* and *tom-1*), *hpo-30* does not appear to affect the secretion of synaptic vesicles. Although these experiments indicate that HPO-30 may not be involved in the retrograde signaling pathway, further experiments delineating the role of HPO-30 in presynaptic cholinergic neurons may help get more insight on possible presynaptic functions of HPO-30 at the NMJ. We further show that NLG-1 levels are negatively regulated by HPO-30 and that *nlg-1;hpo-30* mutants suppress the *hpo-30* mutant phenotype and show behaviors similar to *nlg-1* mutant animals, indicating that NLG-1 is likely to be functioning downstream of HPO-30. Further, our data also suggest a possible association between HPO-30 and the LAChR receptor subunits. Together, our results suggest that HPO-30 negatively regulates NLG-1 levels, which in turn appears to antagonize LAChR clustering at the synapse. Further experiments could allow for more mechanistic insights into the function of HPO-30 and NLG-1 function at the NMJ.

A growing body of evidence suggests that cell adhesion molecules present at the synapse are not merely structural components, but they are actively involved in regulation and modification of synapse function, synapse formation, and synaptic receptor function (for review, see Dalva et al., 2007). The claudin family of proteins are known to interact with the actin cytoskeleton via other proteins (for review, see Sawada et al., 2003). The actin cytoskeleton acts as an important regulator of synapse assembly and organization (for review, see Dillon and Goda, 2005). The postsynaptic receptor organization and maintenance are dependent on number of proteins, including cell adhesion proteins and PDZ domain containing proteins (for review, see Sheng and Hoogenraad, 2007). The PDZ domain containing intracellular C-terminal domain of NLG-1 facilitates the binding of postsynaptic scaffold proteins (Irie et al., 1997; Dresbach et al., 2004). These proteins interact directly or indirectly with the actin cytoskeleton and hence could regulate receptor organization (for review, see Sheng and Kim, 2011). One possible outcome of HPO-30 function via NLG-1 could be modulation of the actin cytoskeleton, which in turn could allow for changes in ion channel localization at the synapse.

The genetic interaction of HPO-30 with the synaptic cell adhesion protein NLG-1 might serve as a link between cell adhesion and the regulation of ion channel. Our results add to the evidence that cell adhesion molecules can have multiple functions at the synapse.
